# Clinical Presentations of *Pseudomonas aeruginosa* Infection in Children with Leukaemia Treated with Chemotherapy—A Series of 5 Cases with Discussion

**DOI:** 10.3390/jcm15218758

**Published:** 2026-11-09

**Authors:** Maciej Niedźwiecki, Janusz Springer, Piotr Czauderna, Mieszko Czapliński, Ninela Irga-Jaworska, Monika Lejman

**Affiliations:** 1 Department of Paediatrics, Haematology and Oncology, Medical University of Gdańsk, 80-211 Gdańsk, Poland; ninela.irga-jaworska@gumed.edu.pl; 2 Division of Preventive Medicine & Education, Medical University of Gdańsk, 80-211 Gdańsk, Poland; janusz.springer@gumed.edu.pl; 3 Department of Surgery and Urology of Children and Adolescents, Medical University of Gdańsk, 80-211 Gdańsk, Poland; piotr.czauderna@gumed.edu.pl; 4 University Clinical Centre, Medical University of Gdańsk, Dębinki 7, 80-952 Gdańsk, Poland; 194058@uck.gda.pl; 5 Independent Laboratory of Genetic Diagnostics, Medical University of Lublin, A. Gębali 6, 20-093 Lublin, Poland; monika.lejman@umlub.pl; 6 Department of Genetic Diagnostics, Children’s University Hospital, A. Gębali 6, 20-093 Lublin, Poland

**Keywords:** ecthyma gangrenosum, *Pseudomonas aeruginosa*, sepsis, acute leukaemia, case series

## Abstract

**Background:** Ecthyma gangrenosum (EG) associated with *Pseudomonas aeruginosa* can be life-threatening in immunocompromised patients with haematological malignancies. Severe neutropenia and antimicrobial resistance complicate treatment, while the roles of surgery and supportive therapies remain uncertain. **Methods:** We retrospectively reviewed five paediatric patients with leukaemia who developed life-threatening *P. aeruginosa* infections during chemotherapy. Cases were identified through a tertiary paediatric oncology centre’s clinical registry and verified using archived medical records. Clinical, microbiological, therapeutic, and outcome data, including surgical interventions, supportive therapies, and chemotherapy interruptions, were analysed. **Results:** All five patients developed severe infections during chemotherapy. EG affected the perineal or genital region in several cases, including two cases of Fournier’s gangrene. Four patients underwent surgical excision or debridement; surgery was not feasible in one because of critical deterioration. All received antimicrobials, with regimens adjusted according to microbiological findings and susceptibility profiles. Supportive treatments included granulocyte colony-stimulating factor, immunoglobulins, and granulocyte transfusions. Two patients survived into adulthood, including one who remained in remission after haematopoietic stem cell transplantation; three died, one from infection-associated multiorgan failure before surgery, one following leukaemia relapse, and one from post-transplant complications. Infection and its treatment substantially delayed or modified chemotherapy. **Conclusions:** EG associated with *P. aeruginosa* during paediatric leukaemia treatment can cause death and disrupt anticancer therapy. Early multidisciplinary assessment, appropriate antimicrobial treatment, and timely surgical management of necrotic tissue should be considered when feasible. Immunomodulatory interventions and discharge between chemotherapy cycles may benefit selected patients, but their efficacy cannot be established from this small retrospective series. Further multicentre studies are needed to clarify optimal management.

## 1. Introduction

Drug-resistant Gram-negative infections are increasingly reported among hospitalized patients. Common pathogens include *Pseudomonas aeruginosa*, *Acinetobacter baumannii*, *Enterococcus faecium*, and Klebsiella pneumoniae [1,2]. In children with acute lymphoblastic leukaemia (ALL) or acute myeloblastic leukaemia (AML), intensive chemotherapy and prolonged, severe neutropenia (absolute neutrophil count < 100 cells/mm^3^ for >7 days) substantially increase infection risk [3]. Central venous catheters and invasive procedures, including lumbar punctures and suprapubic catheterisation, provide additional routes of infection during intensive chemotherapy. Although Gram-positive pathogens account for most infections in this population (57%), Gram-negative infections, which are less frequent, are associated with higher mortality (18%) [4,5]. Poor outcomes are also associated with *P. aeruginosa*, which causes bacteraemia in approximately 15% of oncological patients, and with its high genetic variability, rapid acquisition of resistance to multiple antibiotic classes, including through β-lactamase production and carbapenem resistance, and increasing multidrug resistance [6,7].

*P. aeruginosa* is a Gram-negative bacillus found in moist environments at temperatures of 4–43 °C and at neutral-to-alkaline pH. Its intrinsic resistance mechanisms, tolerance to certain biocides, ability to form biofilms, and capacity to colonise medical equipment and hospital surfaces contribute to the persistence of *P. aeruginosa* in healthcare environments [6,8].

Immunocompetent individuals are generally resistant to *P. aeruginosa*, whereas immunocompromised patients may develop localised infections of the urinary tract, external or middle ear, paranasal sinuses, eyes, or lungs. Pulmonary infections are particularly relevant in mechanically ventilated patients and those with cystic fibrosis [6,9,10,11]. Bone and joint infections may also arise through bacteraemic spread or extension from a local infection [12,13,14].

Neutropenia is a major determinant of host susceptibility. In neutropenic patients, *P. aeruginosa* infection commonly presents as bacteraemia and may progress rapidly to septic shock [15,16]. A characteristic manifestation is ecthyma gangrenosum (EG), in which bacterial invasion of cutaneous and soft-tissue blood vessels causes necrotic skin lesions. EG occurs in patients with malignancies, premature infants, people with diabetes, patients receiving corticosteroids, malnourished individuals, and those with extensive burns [6]. Important risk factors include gastrointestinal colonisation with *P. aeruginosa* and central venous catheter infection. Although EG can affect any skin site, lesions occur most frequently in the anogenital region (57%), limbs (30%), buttocks, and axillae [6]. Treatment is challenging because of increasing antimicrobial resistance and poor antibiotic penetration into necrotic tissue. Accordingly, early excision of necrotic lesions and wide incision of inflamed tissue have been recommended and reported to improve outcomes significantly [16].

Fournier’s gangrene is a distinct form of necrotizing fasciitis affecting the perineal, perianal, and external genital regions, with infection spreading rapidly along fascial planes [17]. While it is a distinct clinical entity from ecthyma gangrenosum, it can frequently be caused by *Pseudomonas aeruginosa* and thus it is included in our article—Cases 1 and 4 suffered from Fournier’s gangrene. In advanced disease, microbiological testing often identifies mixed pathogens, with *P. aeruginosa* among the reported aetiological agents [18]. In addition to antibiotics, supportive treatments for neutropenic oncology patients with EG may include immunoglobulins, granulocyte colony-stimulating factor (G-CSF), and granulocyte transfusions to enhance immune function. Adequate caloric intake and prevention of catabolism through appropriate enteral or parenteral nutrition are also important [19]. Published data indicate that EG treatment fails in approximately 15% of adults with neutropenia [6].

## 2. Case Series

We present five paediatric oncology patients with ecthyma gangrenosum (EG). Although life-threatening *Pseudomonas aeruginosa* infections are well documented in immunocompromised patients, including those undergoing cancer treatment, this case series provides valuable material for clinical education and highlights questions for further research. Specifically, we summarise the available genetic characteristics of the leukaemias and discuss potential host genetic factors that may influence the incidence and severity of ecthyma gangrenosum; we also discuss potential roles of early surgical intervention, immunomodulatory treatments such as granulocyte transfusions, and discharge planning to reduce exposure to nosocomial *P. aeruginosa*.

### 2.1. Methodology of the Study; Case Selection

We searched the registry of patients treated at the Clinic of Paediatrics, Haematology, Oncology and Immunology, University Clinical Centre of Gdańsk, between 2001 and 2020 to identify life-threatening *P. aeruginosa* infections. The search identified 78 patients. Of these, we included those who 1. suffered from leukaemia; 2. developed *P. aeruginosa* infection, and 3. whose infection occurred during chemotherapy. Sex, age, and chemotherapy regimen were not considered in case selection, provided that all 3 criteria were met. As the registry contained only abbreviated medical histories, each potentially eligible case was verified manually using the full records in the Central Archive. Four cases were initially identified; a fifth was added during the first revision round after an additional eligible case was found.

### 2.2. Case 1

A 17-year-old male with AML (FAB classification, type M3) received chemotherapy according to the Interim AML-BFM 1998 protocol and achieved haematological remission after two cycles. Four months after diagnosis, following the third cycle (HAE), he developed agranulocytosis, dysuria, inflammation of the external urethral meatus, and rapidly rising inflammatory markers (CRP 228 mg/L; PCT 22 ng/mL). Despite empirical treatment with piperacillin/tazobactam, netilmicin, and ciprofloxacin, septic shock developed within 24 h, requiring intensive care, vasopressors, intravenous fluids, and immunomodulatory treatment (G-CSF and immunoglobulins).

**Figure 1:**
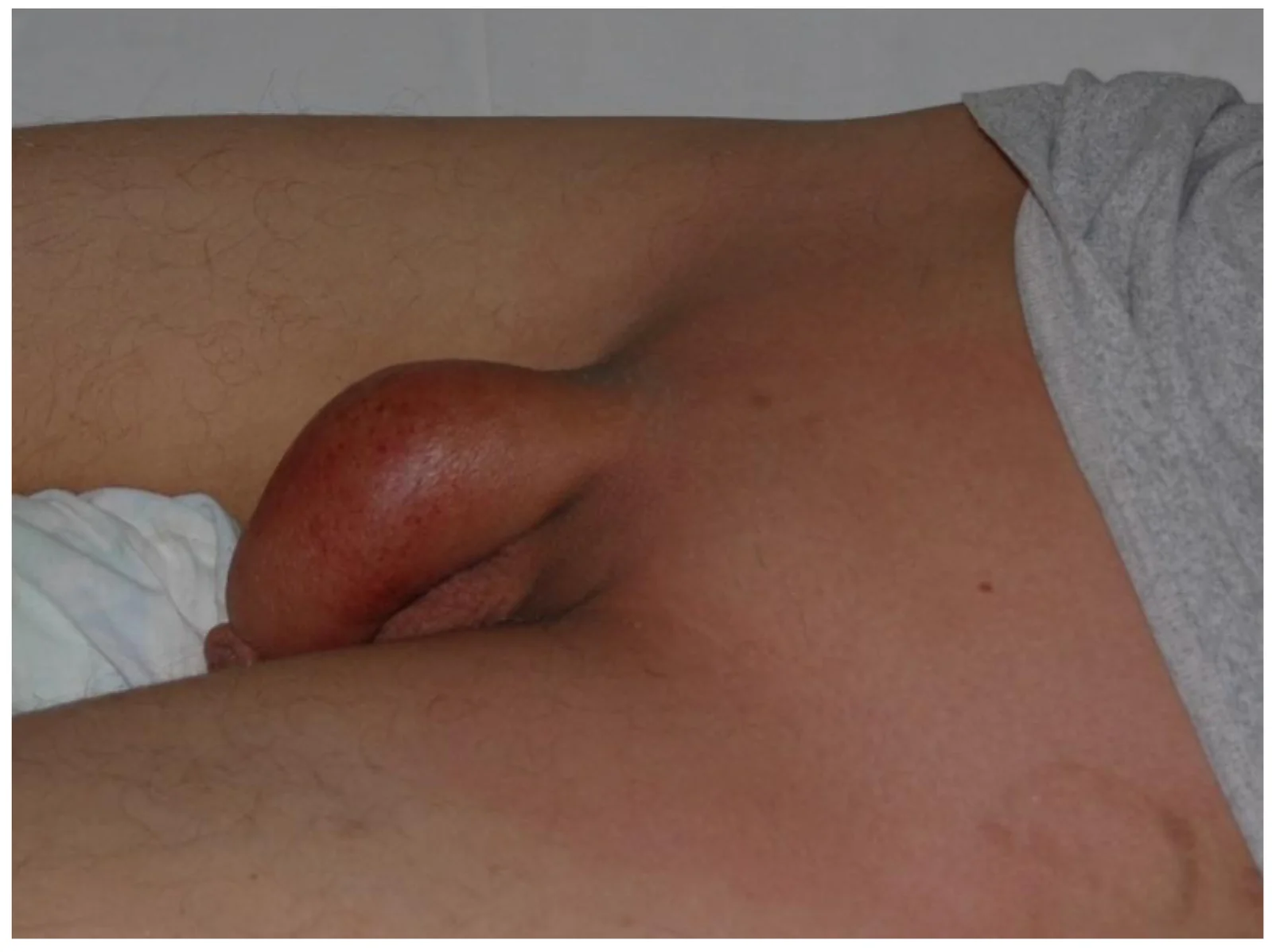
Initial presentation of Fournier’s gangrene in a 17-year-old boy.

Urine and stool cultures were positive for *P. aeruginosa*; stool cultures also yielded vancomycin-resistant *Enterococcus faecium* (VRE), β-haemolytic *Streptococcus*, and *Candida albicans*. Following susceptibility testing, antimicrobial therapy was changed to colistin, teicoplanin, and rifampicin, while amphotericin and caspofungin were added. Septic shock was controlled within 24 h, but inflammation of the groin and penile skin and soft tissues (Figure 1) progressed to extensive necrosis, diagnosed as Fournier’s gangrene. Metronidazole and acyclovir were added for anaerobic and antiviral coverage. Chlorhexidine and octenidine dihydrochloride were applied locally, and a suprapubic urinary catheter was inserted.

Surgical resection of the necrotic tissue was postponed because of the patient’s persistently critical condition and prolonged agranulocytosis. Six days later, shock recurred, and intensive treatment failed to improve his condition. No suitable familial granulocyte donor was identified. Infection progressed, with additional inflammatory foci in the lungs, and the patient died of multiorgan failure.

### 2.3. Case 2

A 9-year-old female with relapsed ALL without central nervous system (CNS) involvement received treatment according to the ALL-REZ BFM 2002 relapse protocol. Two days after the second cycle (F2), she developed pain in the left periauricular region. Laryngological examination revealed a slightly bleeding lesion in the left external auditory canal. As the patient was neutropenic, intravenous cefoperazone was initiated, followed by meropenem and teicoplanin. The next day, erythema, central necrosis, and oedema developed in the left periauricular region, and EG was diagnosed. CRP was 70 mg/L, and blood cultures were positive for *P. aeruginosa*.

Within hours, the patient developed septic shock with hepatic and renal insufficiency, lost consciousness, and was transferred to the intensive care unit. Head computed tomography (CT) revealed CNS haemorrhage associated with disseminated intravascular coagulation (DIC) and possible CNS involvement by the inflammatory process. Although blood pressure and hepatic and renal function improved, the patient remained comatose and required intubation and mechanical ventilation.

**Figure 2:**
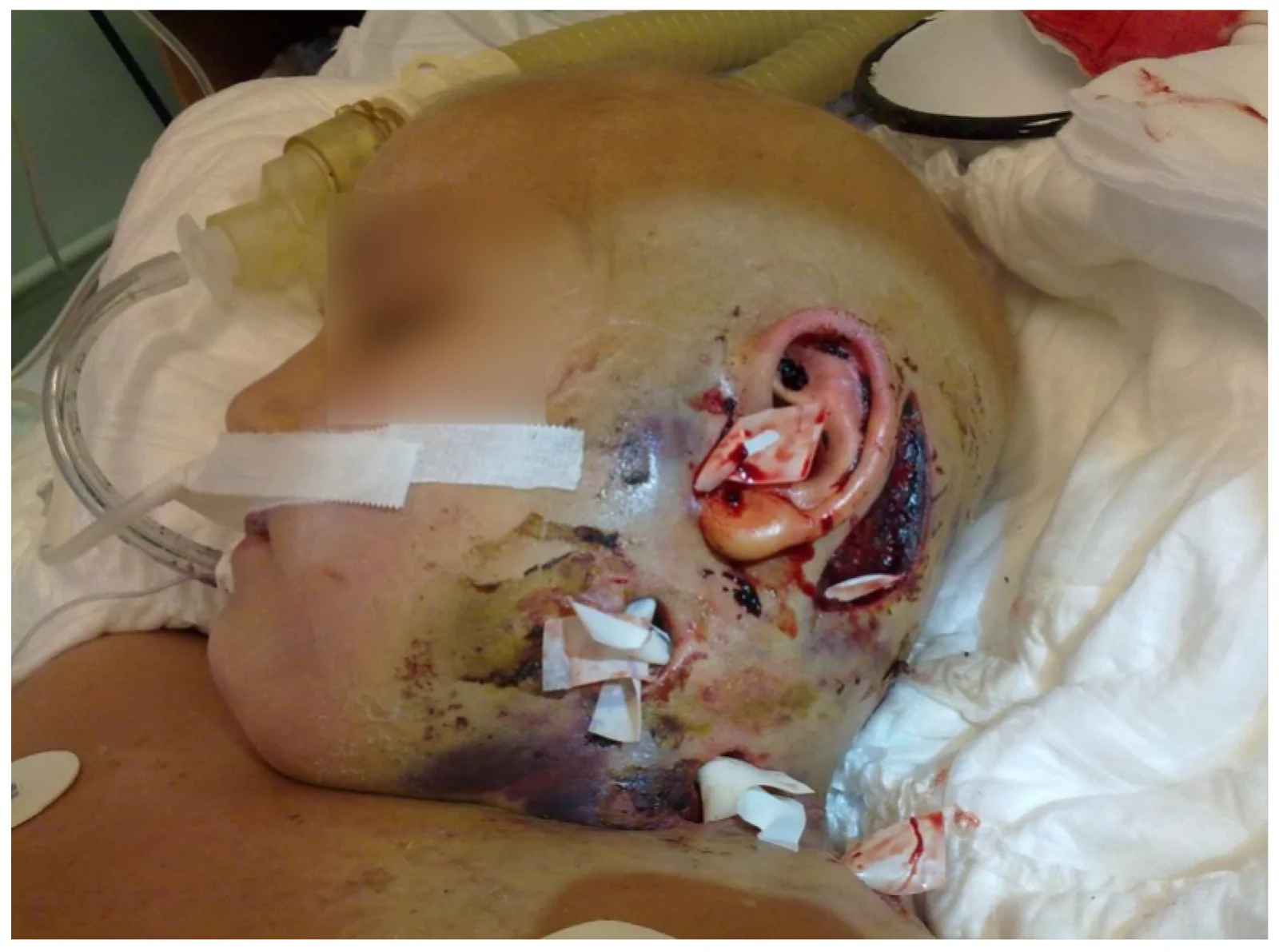
Ecthyma gangrenosum in the periauricular region of a 9-year-old girl. The condition was treated with wide surgical incisions of the inflamed tissue.

Wide debridement and excision of the necrotic tissue were performed (Figure 2). The existing antimicrobial regimen was continued, with amphotericin and caspofungin added, alongside G-CSF, immunoglobulins, and granulocyte transfusions. Over the following week, the patient gradually regained consciousness and was extubated. The skin lesions healed within four weeks, and chemotherapy was resumed at 80% of the planned doses. Because chest CT revealed fungal lesions during the septic episode, antifungal treatment continued until chemotherapy was completed.

Despite severe myelosuppression-related adverse effects occurring until the end of treatment, no further bacterial infections occurred. Treatment was completed with allogeneic haematopoietic stem cell transplantation (HSCT) from an unrelated donor. Extensive rehabilitation enabled recovery of speech and motor function. Four years after transplantation, the patient remained in remission and in good general condition, although with some neurological deficits, which are still present today.

### 2.4. Case 3

An 8-year-old female with relapsed high-risk ALL was treated according to the ALL-REZ BFM 2002 protocol. On the third day of the F2 cycle, she developed agranulocytosis, groin pain, and erythema. As inflammatory markers rapidly increased, cefoperazone and amphotericin were initiated. Blood, urine, and groin swab cultures were positive for *P. aeruginosa*. Despite early antibiotic treatment, septic shock developed within 24 h, accompanied by focal tissue necrosis.

**Figure 3:**
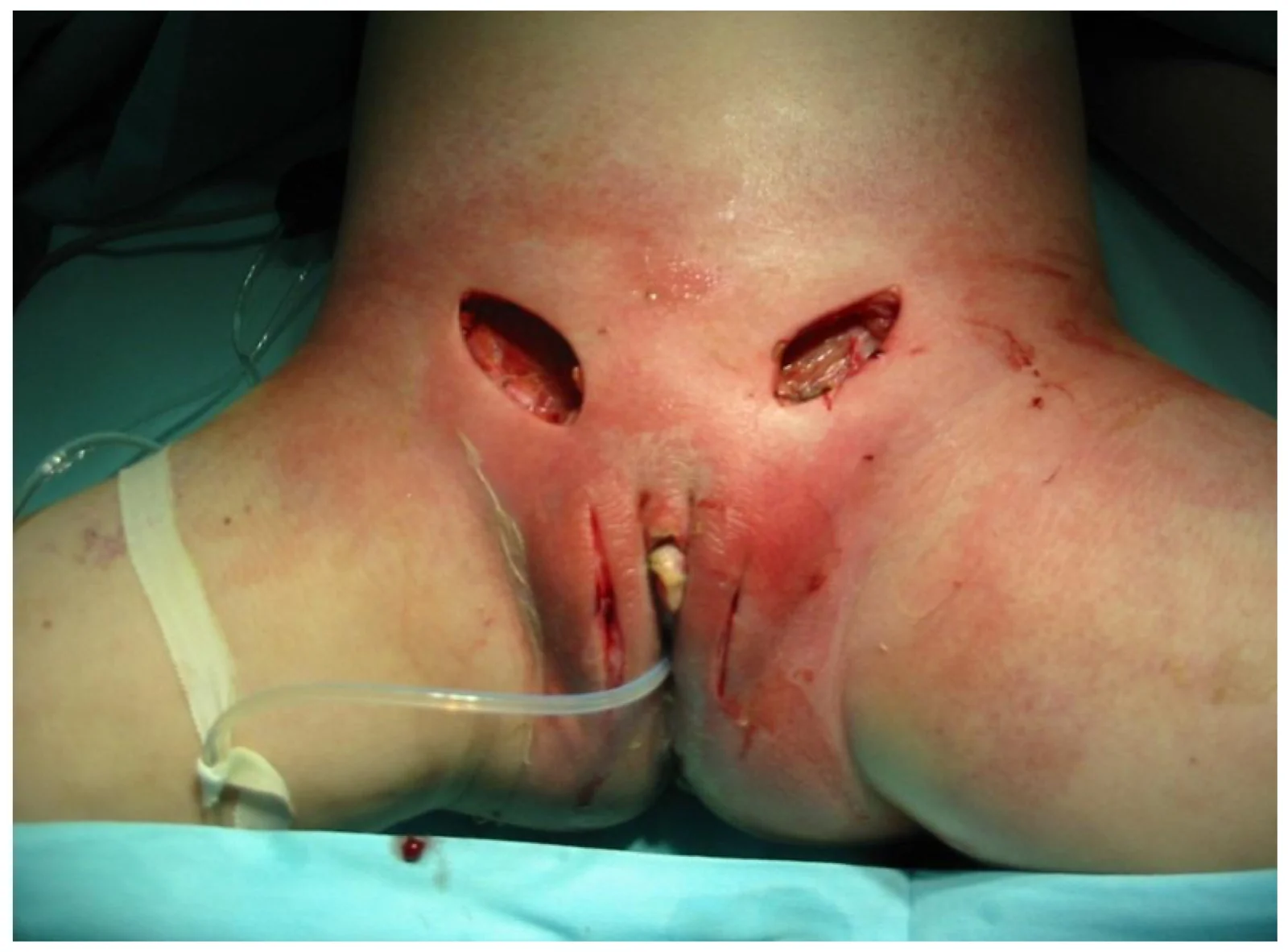
Ecthyma gangrenosum in an 8-year-old girl following surgical excision of necrotic lesions and wide incision of the inflamed region.

Intensive treatment with vasopressors and intravenous fluids stabilised the patient, but her condition remained severe despite G-CSF, immunoglobulins, and granulocyte transfusions. Over the following four days, the lesions spread to the suprapubic region, and the patient was referred for hyperbaric oxygen therapy. Three days later, septic shock recurred. After stabilisation, she underwent surgical resection of the necrotic tissue, with wide incisions of the inflamed areas. Because of the location and extent of the lesions (Figure 3), an ileostomy was required.

The patient gradually improved, and the soft-tissue infection resolved within two weeks. The incisions were subsequently sutured, and the ileostomy was reversed three months after surgery. Chemotherapy resumed six weeks postoperatively at reduced cytostatic doses but was later shortened because of severe toxicity, including myelosuppression, mucositis, and gastrointestinal toxicity. Treatment was completed with allogeneic HSCT. Eight months after transplantation, the patient developed a second ALL relapse and died within the following two months.

### 2.5. Case 4

A 13-year-old boy with bilineal ALL received treatment according to the ALLIC 2002 protocol. From the outset, chemotherapy was poorly tolerated and caused severe adverse effects, including prolonged agranulocytosis, fungal pulmonary infection, and gastrointestinal toxicity. After the HR3 cycle, he developed erythema of the external urethral meatus and oedema of the glans. Broad-spectrum antibiotics (cefepime, teicoplanin, amikacin, and metronidazole) were initiated, together with posaconazole for the ongoing pulmonary fungal infection.

Blood and skin-lesion cultures were positive for *P. aeruginosa*. G-CSF, immunoglobulins, and granulocyte transfusions were added. Despite intensive treatment over the next three days, the inflammation progressed to focal necrosis, and Fournier’s gangrene was diagnosed. The lesions extended to the right inguinal and thigh regions. Five days after infection onset, the patient developed septic shock. Following stabilisation, meatotomy and excision of the necrotic groin tissue were performed. The inflamed skin was incised, and vacuum drainage was applied.

**Figure 4:**
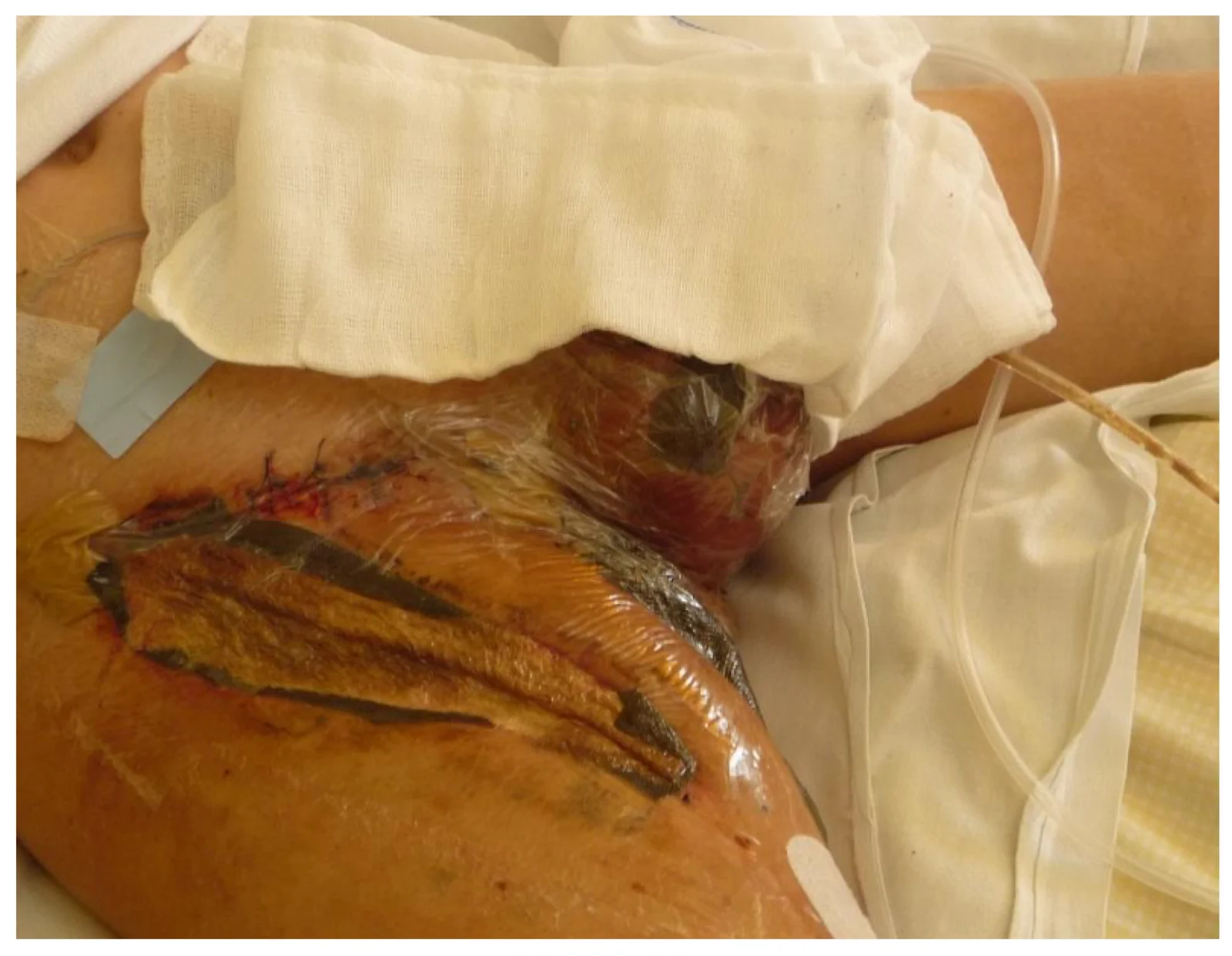
Fournier’s gangrene in a 13-year-old boy treated with wide incisions and local drainage.

Antibiotic therapy was subsequently modified according to susceptibility results, initially using ciprofloxacin, tigecycline, and imipenem, followed by ceftazidime, rifampicin, and chloramphenicol. Caspofungin, amphotericin, and acyclovir were also administered during the course of treatment. Over the next 10 weeks, several additional incisions and extensions of the initial incisions were required (Figure 4). Agranulocytosis persisted for two months. As bone marrow function gradually recovered, the skin lesions also healed.

Chemotherapy was resumed at reduced doses but discontinued because of severe toxicity, including myelosuppression, gastrointestinal toxicity, and mucositis. The patient underwent allogeneic HSCT. Transplantation was complicated by severe fungal pulmonary infection and septic shock, and the patient died of multiorgan failure.

### 2.6. Case 5

An 8-year-old girl with common-type ALL was treated for her first relapse according to the INTREALL SR 2010 protocol. At the end of the SCA7 cycle, she developed groin pain, swelling, and erythema. Initial treatment comprised ciprofloxacin and metronidazole. After microbiological results from the catheter became available, treatment was adjusted, adding meropenem and amikacin. During treatment, *P. aeruginosa* became resistant to all tested antibiotics except colistin, which was therefore added.

**Figure 5:**
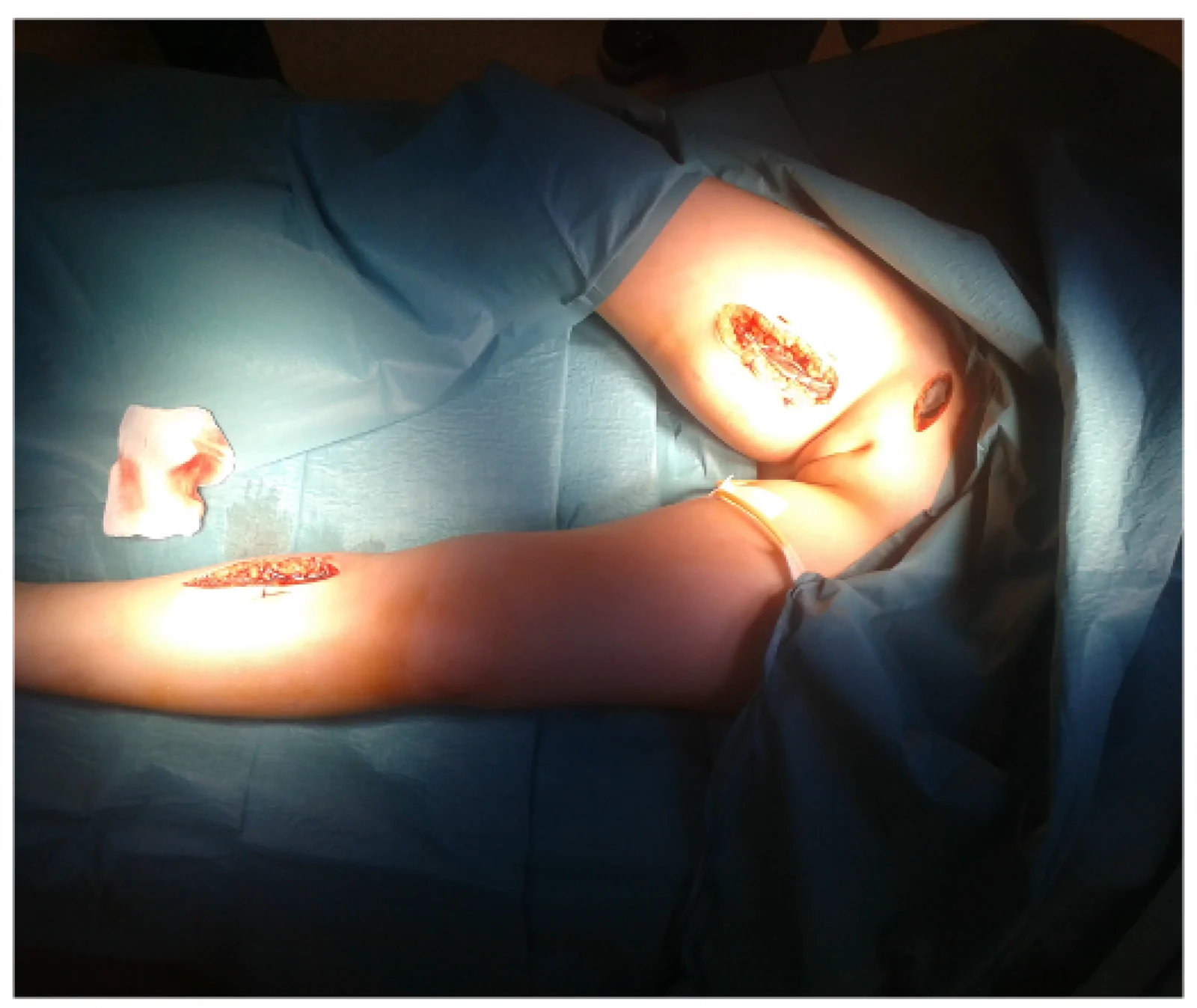
Ecthyma gangrenosum in the groin of an 8-year-old girl, extending to the right lower limb and treated by surgical excision.

The patient underwent seven surgical excisions and abscess debridements (Figure 5). Hyperbaric oxygen therapy was also initiated, and the lesions eventually healed. Chemotherapy was completed, although infection delayed its administration by three weeks. The patient is now 21 years old and has survived into adulthood in remarkably good condition, without reported long-term sequelae.

The summary of all presented cases is shown in Table 1, and the microbiological profile of *P. aeruginosa* isolates is shown in Table 2.

## 3. Discussion and Clinical Implications

### 3.1. P. aeruginosa Infections in Paediatric Oncology and Their Treatment in Light of Current Standards

*P. aeruginosa* is an important opportunistic pathogen in paediatric oncology. Richert-Przygońska et al. [20] analysed data from 17 Polish paediatric oncology centres and six transplant centres. Among approximately 10,000 children treated at these centres between 2014 and 2023 and 1600 children undergoing HSCT, the incidence of *P. aeruginosa* infection was approximately 6–7%; 23% of infections involved bacteraemia. Urinary tract infections were most frequent, followed by skin and soft-tissue infections. Although these infections were often prolonged, recurrent, and difficult to treat, the infection-related mortality was 4.9%. The most frequently used antibiotics were meropenem, ceftazidime, and piperacillin/tazobactam. Resistance rates were substantial, reaching 46.7% for meropenem and 42.2% for piperacillin/tazobactam. Multidrug resistance was identified in up to 38.2% of isolates and was more frequent among HSCT recipients. Colistin was required in 5.3% of cases [20].

Other studies have highlighted the severity and mortality associated with *P. aeruginosa* infections and antimicrobial resistance in children. A Spanish multicentre study of 151 patients reported carbapenem resistance in 29.8% of isolates and 30-day mortality of 23.2% [21]. Chen et al. [22] reported 60-day mortality of 17.3% in a paediatric cohort with *P. aeruginosa* bacteraemia. A study from Queensland reported 10.5% mortality among 261 bloodstream-infection episodes [23]. These figures are not directly comparable because the studies differed in populations, outcomes, and follow-up periods. In particular, mortality from bloodstream infections should not be equated with mortality from all *P. aeruginosa* infections. Nevertheless, they illustrate the clinical importance of these infections. Importantly, *P. aeruginosa*-associated mortality reported in these studies should not be equated with overall mortality in children with leukaemia. In our series, only one death was directly attributable to *P. aeruginosa* infection; of the other two patients who died, one died of HSCT-related complications and one of relapsed leukaemia. Each patient was thus exposed to at least three competing risks of death: infection, treatment toxicity and the underlying disease.

No single guideline specifically addresses all aspects of *P. aeruginosa* infection in paediatric oncology. Current management of such cases in our centre therefore derives from several complementary sources:

- The 2023 guidelines for managing fever and neutropenia in paediatric patients with cancer [24].
- The 2026 IDSA guidance on antimicrobial-resistant Gram-negative infections [25].
- The 2014 IDSA guidelines for skin and soft-tissue infections [26].
- A 2021 publication addressing ecthyma gangrenosum in children with cancer [27].

Together, these sources support a risk-adapted approach to antimicrobial therapy. In children with high-risk febrile neutropenia, intravenous antibiotics active against *P. aeruginosa*, such as piperacillin/tazobactam, cefepime, or a carbapenem, should be initiated promptly. In patients with septic shock, a high risk of antimicrobial resistance, or suspected catheter-related infection, additional agents may be indicated, depending on the clinical presentation and suspected pathogens. These may include an aminoglycoside or, when specifically indicated, vancomycin. Treatment should subsequently be adjusted according to microbiological findings and susceptibility results. Some paediatric oncology protocols recommend continuing two active agents for confirmed *P. aeruginosa* infection [28], although this is not a universal recommendation and should be distinguished from routine empirical combination therapy.

Surgery is not routinely indicated for every infection; its role depends on the presence of a surgically treatable focus, such as an abscess or necrotic tissue in EG. Evidence supporting hyperbaric oxygen (HBO) specifically for EG remains insufficient. Although HBO has been used as an adjunct in selected severe infections, its efficacy in EG has not been established, and it must not delay appropriate antimicrobial treatment or necessary surgery.

### 3.2. Potential Genetic Determinants of Severe Clinical Course in P. aeruginosa Infections

Genetic factors may influence the course of *P. aeruginosa* infection in children with leukaemia at two levels: through the somatic genetic profile of the leukaemia, which determines the risk group and, consequently, the intensity of chemotherapy and the depth and duration of neutropenia, and through constitutional (germline) variation of the host, which may affect drug metabolism, chemotherapy-related toxicity and innate immune defence.

Genetic data obtained during routine diagnostics were available for four of the five patients, and their leukaemias were genetically heterogeneous. In Case 1 (AML), the bone marrow karyotype was normal (46,XY[20]), and neither ETV6::RUNX1 fusion nor FLT3 internal tandem duplication (FLT3-ITD) was detected. In Case 2, B-cell precursor ALL at initial diagnosis showed high hyperdiploidy (51,XXX,+6,+13,+19,+21[7]/46,XX[4]), with four copies of RUNX1 detected by fluorescence in situ hybridisation (FISH), consistent with tetrasomy 21, and without KMT2A rearrangement. High hyperdiploidy is the most common cytogenetic subgroup of childhood B-cell precursor ALL and is generally associated with a favourable prognosis [29]; nevertheless, this patient subsequently relapsed, and genetic data from the time of relapse were not available. In Case 3, the karyotype at relapse was normal (46,XX[10]), and FISH excluded BCR::ABL1 fusion and KMT2A rearrangement. In Case 4 (bilineal ALL), the karyotype revealed a rare balanced translocation, t(9;14)(p22;q13), present in all abnormal metaphases, and a subclone with an additional deletion of the short arm of chromosome 2, del(2)(p13); FLT3-ITD was not detected. This patient also showed a poor early response to treatment, with 71% blasts in peripheral blood on day 8 and 55% blasts in bone marrow on day 15 of induction. No genetic data were available for Case 5.

No recurrent somatic alteration was shared by the patients, and in this small series the genetic subtype of leukaemia does not appear to have determined susceptibility to, or severity of, *P. aeruginosa* infection. Its influence was rather indirect: in Cases 2–5, the infection occurred during intensive relapse or high-risk treatment blocks, which are associated with the most profound and prolonged neutropenia. It should also be noted that genetic diagnostics in these patients were limited to conventional cytogenetics, targeted FISH and PCR, in accordance with the standards of the time. Submicroscopic copy-number alterations and cryptic rearrangements, which are now routinely detected by microarrays or next-generation sequencing, could therefore not be assessed.

Constitutional variants affecting drug metabolism are a well-established cause of excessive treatment-related toxicity in children with leukaemia. Inherited deficiency of thiopurine S-methyltransferase or nudix hydrolase 15, encoded by TPMT and NUDT15, respectively, leads to accumulation of active thioguanine nucleotides and to severe, prolonged myelosuppression in patients receiving standard doses of mercaptopurine or thioguanine [30,31,32]. Loss-of-function NUDT15 variants are common in East Asian and Hispanic populations but rare in Europeans [31]; in Polish patients, TPMT is therefore of greater practical relevance. Similarly, variants in SLCO1B1 influence methotrexate clearance and, consequently, the risk of methotrexate toxicity [33]. As profound and prolonged neutropenia is the main risk factor for *P. aeruginosa* bacteraemia and EG, a pharmacogenetic predisposition to myelotoxicity could contribute both to the development of severe infection and to poor tolerance of subsequent chemotherapy. Notably, Case 4 tolerated chemotherapy poorly from the start of treatment, and in Cases 3 and 4 chemotherapy resumed after the infection had to be shortened or discontinued because of severe toxicity. Pharmacogenetic testing was not part of routine practice when these patients were treated. Pre-emptive TPMT and NUDT15 genotyping, recommended by current guidelines [30], allows dose adjustment before thiopurine exposure and may reduce the risk of life-threatening infectious complications.

Inherited variation in genes of innate immunity may also modulate the course of infection during neutropenia. Children with malignancy carrying MBL2 variants associated with mannose-binding lectin deficiency were reported to have longer febrile neutropenic episodes [34], and combined MBL2 and FCN2 risk genotypes were associated with a higher number of bacterial infections in children with B-cell precursor ALL [35]. However, another study found no association between mannose-binding lectin deficiency and infections during induction therapy [36], so the clinical relevance of these variants remains uncertain. Rarely, unusually severe infections in a child with leukaemia may reflect an inherited syndrome predisposing both to haematological malignancy and to immunodeficiency, such as GATA2 deficiency, which is associated with myelodysplastic syndrome, AML, monocytopenia and severe infections [37], or combined immunodeficiency caused by germline IKZF1 variants [38]. Such conditions should be considered particularly when cytopenias or recurrent infections preceded the diagnosis of leukaemia or when the family history is suggestive.

No constitutional genetic testing was performed in any of our patients, so the contribution of host genetic factors could not be assessed. Retrospective germline analysis would require DNA from a non-haematopoietic source, such as cultured skin fibroblasts, because blood and bone marrow samples obtained before transplantation contain leukaemic cells, and after allogeneic HSCT (Cases 2–4) blood-derived DNA reflects the donor genotype. Prospective studies combining systematic pharmacogenetic and germline analyses with clinical and microbiological data are needed to determine whether host genetic factors contribute to the occurrence and severity of EG in children with leukaemia.

### 3.3. Antimicrobial Therapy, Its Efficiency and Reasons for Possible Failure of Conservative Treatment

Our cases of ecthyma gangrenosum (EG) associated with *P. aeruginosa* infection in children with leukaemia illustrate the severity of these infections, the challenges of treatment, and the potential for poor outcomes. Broad-spectrum antibiotics alone may be insufficient, particularly when additional interventions are required. The effectiveness of antimicrobial therapy in paediatric oncology patients may be influenced by several factors.

Chemotherapy can alter drug metabolism and produce pharmacological interactions that may affect antimicrobial exposure and efficacy. As the infections in our series developed during chemotherapy, these interactions may have influenced treatment outcomes. Furthermore, the underlying leukaemia and profound neutropenia impaired host immune defences, potentially worsening the prognosis despite appropriate antimicrobial therapy.

Although *P. aeruginosa* was initially identified as the causative pathogen, tissue cultures frequently yielded additional organisms, including anaerobes, fungi, and other microorganisms [19,39]. Consequently, antimicrobial regimens also had to provide coverage for these additional pathogens, further complicating treatment and potentially affecting its effectiveness.

Moreover, necrotic tissue may limit antimicrobial penetration, reducing drug exposure at the site of infection. The presence of biofilms and walled-off abscesses may further impede pathogen eradication.

Taken together, these factors may help to explain why none of our patients recovered with conservative treatment using broad-spectrum antibiotics alone. Consistent with previous reports, we consider early surgical excision of necrotic tissue to be an important component of management that may improve infection control and minimise delays in chemotherapy, potentially improving outcomes for the underlying leukaemia [16].

### 3.4. Potential Role of Early Surgical Intervention in EG Treatment

Surgery is an important component of infection management in both immunocompetent and immunocompromised patients; some infections, such as abscesses, primarily require surgical drainage. Surgical approaches have also been described for *P. aeruginosa* infections [16,40,41]. However, questions remain regarding optimal timing and the comparative effectiveness of surgery and conservative treatment.

Muggeo et al. [42] examined severe *P. aeruginosa* infections in children with cancer. Of 38 cases, 24 underwent surgery. Although the study did not establish the superiority of surgical over conservative treatment, it demonstrated that surgery is frequently incorporated into management. Regarding timing, Endorf et al. [43] highlighted the importance of urgent debridement in paediatric necrotising soft-tissue infections. Although their study was not limited to *P. aeruginosa*, it included 338 cases and found a statistically significant survival advantage associated with early debridement. A single procedure may not suffice; repeated interventions may be necessary to control infection, as illustrated by Case 2, in which three operations were required. The need for repeated procedures is also supported by previous studies [41].

While determining whether the patient requires early surgical intervention, several important points need to be considered. Haemodynamic stability must be ensured—if the patient is in septic shock they need to be stabilised first before surgery can take place. It is because of this reason that surgery could not be performed in our 1st case. The degree of neutropenia plays an important role as well—severe and long-lasting neutropenia has a direct negative influence on patients’ ability to heal, and potential infections with nosocomial pathogens can be acquired during surgery. On the other hand, rapid progression of infection with expansion of abscesses is an indication for surgical work-up.

In summary, surgery is commonly used to treat EG, and early intervention and repeated procedures, when necessary, may contribute to infection control. In our series, early surgery was associated with favourable outcomes in Cases 2 and 5, whose patients remain alive. However, this association does not establish causality. Further studies are needed to compare surgical and conservative approaches, as randomised trials establishing the superiority of either strategy are currently lacking.

### 3.5. The Potential Role of Granulocyte Transfusions in the Therapy of EG

At our centre, two approaches are routinely used to support immune recovery in neutropenic patients. The first is the administration of filgrastim, a granulocyte colony-stimulating factor (G-CSF), to promote neutrophil recovery. Neutropenia in our patients may result either from the underlying disease or from the chemotherapy administered, and filgrastim is used to support the restoration of white blood cell (WBC) counts. The second approach is granulocyte transfusion, usually using granulocytes obtained from family donors. These transfusions can increase circulating white blood cell counts more rapidly and are particularly important in patients with profound or prolonged neutropenia.

Granulocyte colony-stimulating factor (G-CSF) and granulocyte–macrophage colony-stimulating factor (GM-CSF) have been discussed as potential supportive treatments for EG. Pulido et al. [44] described EG in an immunocompromised infant with leukaemia who had received filgrastim (G-CSF), although neutrophil recovery was not immediate. Bécherel et al. [45] reported the use of GM-CSF in the management of severe EG associated with myelodysplastic syndrome. At our centre, G-CSF is routinely used to support the recovery of WBC counts. However, in neutropenic children with life-threatening infections, granulocyte transfusions may be preferred when a more immediate increase in circulating granulocytes is required. Therefore, the remainder of this subsection focuses on their potential role in the management of EG.

Several reports have described favourable outcomes following immunostimulatory treatment in patients with EG. Wuyts et al. [46] reported a child with recurrent EG associated with underlying cyclic neutropenia who experienced fewer infections and no further EG episodes following G-CSF treatment. Although the patient did not have leukaemia, this case suggests that G-CSF may help prevent EG recurrence in patients with underlying neutropenia. Favourable outcomes following granulocyte transfusions for EG have also been reported by Mellouli et al. [47]. Their report described a child with leukocyte adhesion deficiency type 1, a primary immunodeficiency, who developed ecthyma gangrenosum associated with *Fusarium solani* and improved following combined treatment that included granulocyte transfusions. These findings suggest a potential role for granulocyte transfusions in selected patients with severe infections and impaired neutrophil-mediated host defence, although the evidence remains limited to individual reports.

In our series, infections developed during severe, prolonged neutropenia, and three patients (Cases 2, 3, and 4) received granulocyte transfusions alongside broad-spectrum antibiotics. All three recovered from the acute infection, although their longer-term outcomes differed. Despite persistent local inflammation, systemic deterioration was temporarily controlled, allowing time for surgical intervention. Clinical improvement was observed, although it was not always reflected in laboratory parameters. In some cases, including Case 2, repeated transfusions were required to achieve a favourable clinical response.

Sever et al. [48] investigated the effects of granulocyte transfusions on inflammatory markers in a retrospective study involving 312 transfusions in approximately 50 children. Despite the methodological limitations inherent in the retrospective design, the study demonstrated a decrease in C-reactive protein (CRP) levels during treatment. The increase in neutrophil counts was transient, with levels generally returning towards baseline within approximately 24 h. Infection-related mortality in the study cohort was 17%, while transfusion-related adverse events occurred in 3% of transfusions.

In light of these findings, granulocyte transfusions could be considered early in selected cases, particularly when temporary clinical stabilisation is needed before planned surgical intervention. Nevertheless, our observations cannot establish their efficacy. Further prospective, controlled studies are needed to clarify the role of granulocyte transfusions and other immunostimulatory therapies in the treatment of EG.

There are some limitations of granulocyte transfusions and the reasons for their potential lack of efficacy also warrant consideration. Although we observed an association between granulocyte transfusion and favourable outcomes in several patients, Case 5 recovered without receiving granulocyte transfusions. It therefore remains uncertain to what extent the improvements observed in our patients can be attributed to granulocyte transfusions alone, particularly given that treatment also included broad-spectrum antibiotics, surgical interventions, and, in selected cases, hyperbaric oxygen therapy or vacuum-assisted closure (VAC). Our case series does not allow us to determine the independent contribution of granulocyte transfusions to clinical outcomes.

### 3.6. Discharge as a Possible Strategy for Reducing Nosocomial Infections

Hospital wards can harbour nosocomial pathogens with substantial antimicrobial resistance, posing particular risks in paediatric oncology. Molecular techniques, including next-generation sequencing (NGS), can help to characterise organisms present in hospital environments. Comar et al. [49], for example, analysed microbial genetic material collected from several hospital departments, including paediatric oncology. Potential reservoirs may include small installations and contaminated environmental surfaces [50]; their presence can contribute to outbreaks with serious consequences [51].

Discharging children and sending them home between chemotherapy cycles may reduce their exposure to hospital-associated pathogens. Getz et al. [52] compared early discharge with prolonged hospitalisation and reported slightly lower mortality in the early-discharge group. Bacteraemia occurred in 23% of children who were discharged early, compared with 29% of those hospitalised for longer. Inter-cycle discharge was also used in our patients. We therefore suggest that discharge home between chemotherapy cycles may be considered for appropriately selected children to reduce hospital exposure. This approach must be balanced against clinical stability, treatment requirements, and the feasibility of safe outpatient monitoring. Strict adherence to antisepsis protocols is also essential. Detailed ward-level epidemiological surveillance may facilitate early implementation of measures to control or eradicate *P. aeruginosa* [53,54].

### 3.7. Effect of EG on Chemotherapy Administration in Our Patients

Severe infections and their complications substantially disrupted the administration of chemotherapy in all five patients. The consequences ranged from discontinuation of chemotherapy because of persistent neutropenia to delays of up to 2.5 months in subsequent cycles. In three patients, treatment delays were accompanied by dose reductions to 80% of the planned doses or other dose modifications, whereas one patient experienced a three-week delay without subsequent dose reduction. These interruptions and modifications may have complicated the delivery of optimal antileukaemic treatment. However, although relapse occurred during the subsequent clinical course in some patients, our small case series does not allow us to establish whether treatment delays or dose reductions contributed to disease progression or whether altered cytostatic drug metabolism played any role, especially as the final outcome could be affected by disease risk, previous relapse, treatment toxicity and adverse drug reactions, and other clinical factors like cachexia may also have contributed.

### 3.8. Limitations of This Case Series

This case series has several limitations that restrict the generalisability of its findings and their direct application to clinical practice. First, the sample size was very small, comprising only five patients who met the inclusion criteria. The retrospective case-series design further limits the completeness and consistency of the available data and introduces a risk of selection bias, as the cases included may not be representative of all paediatric patients with EG, as well as potential reporting bias related to the availability and completeness of archived medical records. Second, this was a single-centre study, and treatment decisions were influenced by local resources, departmental practices, and individual physicians’ experience and preferences. Our institution is a paediatric oncology referral centre within a multidisciplinary hospital, with other specialties readily available; this may have affected the feasibility and timing of surgical interventions and limits the generalisability of our findings to other clinical settings.

Comparisons between cases and with published studies are further complicated by heterogeneity in patients’ leukaemia status, clinical presentation, and treatment. Patients had different *P. aeruginosa* isolates and antimicrobial susceptibility profiles, necessitating different antimicrobial regimens. Additional pathogens frequently required further antimicrobial coverage. Treatment protocols also varied between patients, including differences in antimicrobial selection and the timing of surgical intervention. Consequently, the individual contributions of antimicrobial therapy, surgery, and other adjunctive treatments cannot be readily disentangled. Furthermore, the retrospective nature of the study meant that management was based on the clinical practices and therapeutic options available at the time. A prospective study conducted today might incorporate interventions that were less commonly used 10–15 years ago, such as vacuum-assisted wound therapy (VAC). A prospective protocol could also facilitate more consistent documentation and greater standardisation of treatment across patients.

Finally, the absence of a comparator group and the lack of prospective allocation to surgical or conservative treatment preclude reliable comparisons between treatment strategies. Given the small sample size, heterogeneous clinical and treatment characteristics, and retrospective design, we cannot establish causal relationships between specific interventions and outcomes or determine the superiority of one treatment strategy over another. These limitations should be considered when interpreting the observed outcomes. Further multicentre studies with larger patient cohorts, systematic data collection, and appropriate comparison groups are needed to clarify the roles of early surgery, antimicrobial treatment, immunomodulatory treatments, and other adjunctive interventions in paediatric EG.

## 4. Conclusions

In summary, this case series presents 5 cases of children with leukemia undergoing chemotherapy, in whom a life-threatening infection with *P. aeruginosa* developed during the course of chemotherapy. We present possible approaches, including multimodal antimicrobial therapy, surgical interventions, immunomodulatory treatment and discharge between cycles to minimise the risk of nosocomial infections. All these approaches can be considered while treating ecthyma gangrenosum, but more studies are needed to establish their place in combined, multidisciplinary management strategy.

## Figures and Tables

**Table 1:** Clinical characteristics and outcomes of the five cases.

	Case 1	Case 2	Case 3	Case 4	Case 5
Age	17	9	8	13	8
Leukaemia subtype	AML M3	ALL, common	B-ALL, common	Bilineal ALL	ALL, common
Risk group	S2	High-risk	S2	High-risk	Standard-risk
Disease status	Primary, remission	1st relapse, without CNS involvement	1st relapse	Primary	1st relapse
Chemotherapy protocol & cycle	Interim AML-BFM 1998, 3rd cycle HAE	ALL-REZ BFM 2002, F2 cycle	ALL-REZ BFM 2002, F2 cycle	ALLIC 2002, HR3 cycle	INTREALL SR 2010, SCA7
Severity & duration of neutropenia	<200, 19 days	<500, 21 days	<200, 15 days; before infection she had 200,000 blasts/uL	<200, 2 months in intervals	<200, 9 days
Initial clinical presentation	Dysuria, penile swelling	Auricular pain, bleeding from the ear	Pain and erythema of the groin	Erythema of external urethral meatus, oedema of glans penis	Oedema of right femoral region and groin, fever
Interval from symptom onset to diagnosis	7 days	3 days	1 day	1 day	1 day
Interval from diagnosis to surgical intervention	Not applicable; patient’s condition was too poor for surgery	3 days; three surgeries, followed by further debridement	9 days	5 days	6 days
ICU admission	No	Yes, for 28 days	Yes, for 20 days	No	No
Chemotherapy interruption/dose reduction	Chemotherapy discontinued; neutropenia prevented the next cycle	1-month delay; subsequent doses reduced to 80%	5-weeks delay in administration of the next cycle, doses reduced to 80%	2.5-month delay; subsequent dose reduction required	3-week delay to the next cycle, no dose reduction
HSCT	No	Yes, 7 months later	Yes, 6 months later	Yes, 6 months later	No
Final outcome	Death 19 days after symptom onset, 5 months after ALL diagnosis	Survived into adulthood; currently 25 years old, with several neurological deficits	After HSCT one more recurrence, this time fatal (death after 2 months)	Death after HSCT due to complications	Survived till adulthood, currently 21 years old, no disease consequences
Granulocyte infusion	No	Yes, 3 times in ICU	Yes, 2 times	Yes, 2 times	No
Cytogenetic and molecular findings	46,XY[20]; ETV6::RUNX1 neg.; FLT3-ITD neg.	51,XXX,+6,+13,+19,+21[7]/46,XX[4]; tetrasomy 21 (FISH); KMT2A rearrangement neg. |	(at relapse): 46,XX[10]; BCR::ABL1 neg.; KMT2A rearrangement neg. |	46,XY,t(9;14)(p22;q13)[16]/46, idem,del(2)(p13)[4]; FLT3-ITD neg.	not available

**Table 2:** Microbiological characteristics of the five cases.

	Case 1	Case 2	Case 3	Case 4	Case 5
Source of microbiological specimen	Urethral swab	Blood sample, catheter	blood sample	Blood sample, urine sample, catheter	Blood sample, catheter
Microorganisms detected	*P. aeruginosa* (++), B-hemolytic Streptococci (++), *E. faecium* VRE (++), *Candida albicans* (++)	*E. coli*, *P. aeruginosa*	*P. aeruginosa*	*P. aeruginosa*	*P. aeruginosa*
Resistance profile	*P. aeruginosa*: susceptible to ciprofloxacin and colistin; resistant to piperacillin, tazobactam, cefepime, ceftazidime, imipenem, meropenem, amikacin, cefoperazone, sulbactam. Streptococci susceptible to piperacillin. *E. faecium*: susceptible to linezolid; resistant to piperacillin, tazobactam, cefepime, ceftazidime, imipenem, meropenem, amikacin, cefoperazone, sulbactam.	Both isolates susceptible to piperacillin + tazobactam, cefepime, ceftazidime, imipenem, meropenem, amikacin, ciprofloxacin, cefoperazone, sulbactam; resistant to ampicillin, piperacillin, cephazolin	*P. aeruginosa* susceptible to cefepim, ceftazidin, imipenem, meropenem, amikacin; resistant to piperacillin, tazobactam, gentamycin, ciprofloxacin, cefoperazone, sulbactam	*P. aeruginosa* susceptible to amikacin, cefepime, cefoperazone + sulbactam, meropenem, ciprofloxacin, piperacillin + tazobactam, ceftazidime, imipenem; no resistance detected	*P. aeruginosa* susceptible to piperacillin, ceftazidime, cefepime, imipenem, meropenem, gentamycin, amikacin, ciprofloxacin
Confirmation with other techniques—genetic, histopathological	No	No	No	No	No
Other microbiological specimens	Prepuce swab, urine, catheter	Repeated blood samples, wound swabs, tracheostomy pipe swabs	Nasal swab, urine sample, urethra swab	Prepuce swab	Urine sample, abscess swab, pharyngeal swab
Concordance with primary finding	Yes	Yes	Yes	No—CRPA detected, reduced susceptibility to imipenem	No—change in resistance profiles; resistant to piperacillin, tazobactam, ceftazidim, cefepime, cefoperazone, imipenem, meropenem, amikacin, and ciprofloxacin; sensitive only to colistin
Other microorganisms isolated from additional specimens	No	No	Yes—*S. aureus*, *Candida albicans*	No	Yes—*Candida albicans*
Initial antimicrobial therapy	Piperacillin, tazobactam, linezolid, amphotericin B, netromycin, cotrimoxazole	Teikoplanin, sulperazone	Sulperazone, amphotericin B	Cefepime, teikoplanin, amikacin, posaconazole	Ciprofloxacin, metronidazole
Modification after microbiology results	Added ciprofloxacin, tigecyclin, colistine, linezolide, aciclovir; removed piperacillin, tazobactam, netromycin	Added meropenem, caspofungin	Added amikacin, removed sulperazon, added meropenem, metronidazol	Added metronidazole	Added meropenem, amikacin, metronidazole
Further changes	One week later, metronidazole, caspofungin, teikoplanin added	After return from ICU: added linezolid, cefepim, fluconazole; removed meropenem, caspofungin	Added vancomycin, 3 weeks after being left only on trimetoprim + sulfametoksazole	Added ciprofloxacin, tigecyclin, imipenem, amphotericin B; removed teikoplanin, amikacin	Added fluconazole, added colistin

Note: All antimicrobial agents were administered according to the standard dosages as indicated in the Cards of Characteristic of Medical Product, with adjustments accounting for the patients’ weight and renal function where applicable.

## Data Availability

Basic data is provided within the manuscript. Other data is available on request. In order to obtain this data, contact the corresponding author.

## Abbreviations

The following abbreviations are used in this manuscript:

ALL	Acute lymphoblastic leukaemia
ALL-REZ BFM	Acute lymphoblastic leukaemia relapse protocol of the Berlin–Frankfurt–Münster (BFM) study group
ALLIC	Acute Lymphoblastic Leukaemia Intercontinental protocol
AML	Acute myeloid leukaemia
AML-BFM	Acute Myeloid Leukaemia Berlin–Frankfurt–Münster protocol
B-ALL	B-cell acute lymphoblastic leukaemia
BFM	Berlin–Frankfurt–Münster
CNS	Central nervous system
CRP	C-reactive protein
CRPA	Carbapenem-resistant *Pseudomonas aeruginosa*
CT	Computed tomography
DIC	Disseminated intravascular coagulation
EG	Ecthyma gangrenosum
FAB	French–American–British classification
G-CSF	Granulocyte colony-stimulating factor
HAE	Homoharringtonine, cytarabine (Ara-C), and etoposide
HBO	Hyperbaric oxygen
HSCT	Haematopoietic stem cell transplantation
ICU	Intensive care unit
IDSA	Infectious Diseases Society of America
INTREALL SR	International study protocol for relapsed acute lymphoblastic leukaemia, standard-risk group
NGS	Next-generation sequencing
PCT	Procalcitonin
SR	Standard risk
VAC	Vacuum-assisted closure (vacuum-assisted wound therapy)
VRE	Vancomycin-resistant *Enterococcus*
